# Differential Effects of Insulin-Like Growth Factor Binding Protein-6 (IGFBP-6) on Migration of Two Ovarian Cancer Cell Lines

**DOI:** 10.3389/fendo.2014.00231

**Published:** 2015-01-05

**Authors:** Zhiyong Yang, Leon A. Bach

**Affiliations:** ^1^Department of Medicine (Alfred), Monash University, Prahran, VIC, Australia; ^2^Department of Endocrinology and Diabetes, Alfred Hospital, Melbourne, VIC, Australia

**Keywords:** insulin-like growth factor, insulin-like growth factor binding protein-6, ovarian cancer, migration, MAP kinase

## Abstract

**Introduction:** IGFBP-6 inhibits angiogenesis as well as proliferation and survival of rhabdomyosarcoma cells. However, it promotes migration of these cells in an IGF-independent manner. The IGF system is implicated in ovarian cancer, so we studied the effects of IGFBP-6 in ovarian cancer cells.

**Methods:** The effects of wild type (wt) and a non-IGF-binding mutant (m) of IGFBP-6 on migration of HEY and SKOV3 ovarian cancer cells, which, respectively, represent aggressive and transitional cancers, were studied. ERK and JNK phosphorylation were measured by Western blotting.

**Results:** IGF-II, wt-, and mIGFBP-6 each promoted SKOV3 cell migration by 77–98% (*p* < 0.01). In contrast, IGF-II also increased HEY cell migration to 155 ± 13% of control (*p* < 0.001), but wt-IGFBP-6 and mIGFBP-6 decreased migration to 62 ± 5 and 66 ± 3%, respectively (*p* < 0.001). In these cells, coincubation of IGF-II with wt but not mIGFBP-6 increased migration. MAP kinase pathways are involved in IGFBP-6-induced rhabdomyosarcoma cell migration, so activation of these pathways was studied in HEY and SKOV3 cells. Wt and mIGFBP-6 increased ERK phosphorylation by 62–99% in both cell lines (*p* < 0.05). Wt-IGFBP-6 also increased JNK phosphorylation by 139–153% in both cell lines (*p* < 0.05), but the effect of mIGFBP-6 was less clear. ERK and JNK inhibitors partially inhibited the migratory effects of wt and mIGFBP-6 in SKOV3 cells, whereas the ERK inhibitor partially restored wt and mIGFBP-6-induced inhibition of HEY cell migration. The JNK inhibitor had a lesser effect on the actions of wtIGFBP-6 and no effect on the actions of mIGFBP-6 in HEY cells.

**Conclusion:** IGFBP-6 has opposing effects on migration of HEY and SKOV3 ovarian cancer cells, but activates MAP kinase pathways in both. Delineating the pathways underlying the differential effects on migration will increase our understanding of ovarian cancer metastasis and shed new light on the IGF-independent effects of IGFBP-6.

## Introduction

The insulin-like growth factor (IGF) system has a key role in normal growth and development (1). IGF-I is regulated by growth hormone and mediates many of its actions, whereas both IGF-I and IGF-II stimulate proliferation, survival, and migration of many cell types. Dysregulation of the IGF system is implicated in many disease processes including cancer, and there has been considerable interest in recent years in developing IGF inhibitors as therapeutic agents (2). However, clinical trials of these inhibitors has been disappointing, and current strategies include understanding mechanisms of resistance as well as finding biomarkers of IGF responsiveness that will permit optimized targeting of these agents.

Insulin-like growth factor actions are regulated by a family of six high affinity binding proteins (IGFBP 1–6) (3, 4). Of these, IGFBP-6 is characterized by a 50-fold binding preference for IGF-II over IGF-I, conferring specificity in inhibiting IGF-II actions (5–7). Most IGFBPs also have IGF-independent actions, and we have shown in recent years that IGFBP-6 inhibits angiogenesis (8) as well as promoting migration of rhabdomyosarcoma and colon cancer cells in an IGF-independent manner (9, 10). Further, we identified cell surface prohibitin-2 as a protein that binds IGFBP-6 and is required for its effects on cell migration (11).

Ovarian cancer is the second most common gynecological malignancy after uterine cancer but the most common cause of gynecological cancer death in Australia (12). In the United States, ovarian cancer is the eighth most common cancer and the fifth leading cause of cancer death in women (13). The diagnosis of ovarian cancer is often delayed because it presents with non-specific symptoms, so that it has metastasized at presentation. Because of this, 5-year-survival is 43%. The IGF system is implicated in ovarian cancer, and both IGF ligands and the IGF-I receptor are expressed in this malignancy (14). In particular, *IGF2* gene expression was 300-fold higher in ovarian cancer tissue than in normal ovarian tissue and expression was higher in advanced stage poor prognosis cancers (15). IGFs have also been shown to stimulate ovarian cancer cell invasion, proliferation, and angiogenesis (16).

IGFBP-6 is commonly expressed at low levels in ovarian cancers (17). A single microarray study showed that IGFBP-6 mRNA levels were lower in ovarian cancer tissue compared with non-cancerous tissue (18); this may reflect derepression of IGF-II action by decreased IGFBP-6, but levels were not confirmed in an independent assay. Small studies of IGFBP-6 in serum of patients with ovarian cancer are inconsistent (19, 20), which may reflect methodological differences. The aim of this study was to determine the effects of IGFBP-6 on migration of HEY, SKOV3, and OVCAR-3 ovarian epithelial cancer cells, which, respectively, represent aggressive, transitional, and less aggressive tumors.

## Materials and Methods

### Reagents

Wild-type (wt) IGFBP-6 and a non-IGF-binding mutant (Pro^93^/Ala, Leu^94^/Ala, Leu^97^/Ala, Leu^98^/Ala) were expressed as His_6_-tagged proteins in *E. coli*, purified by Ni-NTA chromatography, dialyzed against cell culture medium, and then stored in aliquots at −80°C as previously described (9). Phosphatase inhibitor mixtures 1 and 2 and signaling pathway inhibitors PD98095 (for ERK1/2) and SP600125 (for JNK) were from Sigma-Aldrich. Complete EDTA-free protease inhibitor mixture tablets were from Roche Applied Science. Antisera to phospho-ERK1/2 (Thr-204/Tyr-204), phospho-JNK (Thr-181/Tyr-185), total ERK1/2 and JNK, and horseradish peroxidase-conjugated donkey anti-rabbit IgG were from Genesearch (Arundel, Australia). β-actin and PHB2 antisera were from Millipore (North Ryde, Australia). SeeBlue Plus2 molecular weight markers were obtained from Invitrogen.

### Cell culture

HEY, SKOV3, and OVCAR-3 cells, which, respectively, represent aggressive, transitional, and less aggressive human ovarian cancers (21), were used in the study. HEY cells are mostly mesenchymal with a typical molecular signature (N-cadherin and vimentin positive, E-cadherin negative). SKOV3 cells are moderately epithelial, with a flattened cobblestone appearance but a mesenchymal molecular signature (N-cadherin and vimentin positive, E-cadherin negative). OVCAR-3 cells are slowest growing and are epithelial by morphology and their molecular signature (E-cadherin positive, vimentin negative). All cell lines were cultured in a 1:1 mixture of Medium 199 and MCDB 105 (Sigma-Aldrich), supplemented with 2 mM l-glutamine, 100 units/ml penicillin, 100 μg/ml streptomycin, 0.25 μg/ml amphotericin B, and either 10% heat inactivated bovine calf serum (complete culture medium, CCM) or 0.05% BSA (serum-free medium, SFM). Cells were seeded at 37°C, 5% CO_2_ for 24–48 h until they reached 70~80% confluence, followed by serum starvation for 16 h, and then subjected to treatments for various times as specified, without or with IGFBP-6 (wt or mutant, 1 μg/ml), IGF-II (100 ng/ml), and/or pathway inhibitors (30 μM).

### Cell proliferation assay

Cell number was measured after 72 h in serum-free medium using an MTT assay as previously described (22).

### Cell migration assay

Migration assays were performed using a 48-well microchemotaxis chamber (Neuroprobe, Cabin John, MD) as described previously (9). Wt or mIGFBP-6 (1 μg/ml) or IGF-II (100 ng/ml) in SFM were added to the bottom well of the chamber. Polycarbonate filters (12 μm pore size, coated with 100 μg/ml gelatine in 10 mM acetic acid at room temperature for 30 min) were placed over the bottom wells, and cells (5 × 10^4^ cells/well) in SFM were seeded in the top wells. In some experiments, pathway inhibitors PD98095 or SP600125 (30 μM) in SFM were added to both bottom and top chambers. After 16 h, the filter was removed, fixed with 100% methanol for 5 min, and stained with 0.5% crystal violet, 50% methanol for 20 min. The filter was then washed in water to remove excess dye and unbound cells. After mounting the filter on a glass slide, non-migrated cells on the top side of the filter were completely removed. Three random fields per well were photographed, and migrating cells were counted using ImageJ software (National Institutes of Health, Bethesda, MD, USA)^1^. Each data point represents four to seven replicates.

### Phosphorylation assays

Phosphorylation of MAP kinase pathway proteins was examined by Western blotting analysis. After treatment with wt- or mIGFBP-6 for 10 min, cells were washed with cold PBS and lysed in 10 mM Tris–HCl (pH 8.0), 0.1 M NaCl, 1 mM EDTA, 1% Triton X-100 containing protease inhibitors. Lysates were centrifuged at 13,000 rpm for 5 min, supernatants collected and protein concentration determined. Proteins (60 μg/lane) were separated by SDS-10% PAGE and transferred onto nitrocellulose membranes. Western blotting analyses were performed using antibodies against phospho-ERK1/2 (1:2,000) or phospho-JNK (1:1,000). Following an enhanced chemiluminescence reaction, membranes were exposed to X-ray film. The membranes were then reprobed with antisera to total ERK1/2 (1:2,000) or JNK (1:1,000), and finally β-actin (1:10,000). Band intensities were quantitated using ImageJ software.

### Statistics

Results are shown as mean ± SEM of three to nine independent experiments as indicated. Data were analyzed by one-way or two-way ANOVA as appropriate followed by Fisher’s PLSD test to compare individual treatments.

## Results

### IGFBP-6 has differential effects on migration of SKOV3 and HEY ovarian cancer cells

**Figure 1:**
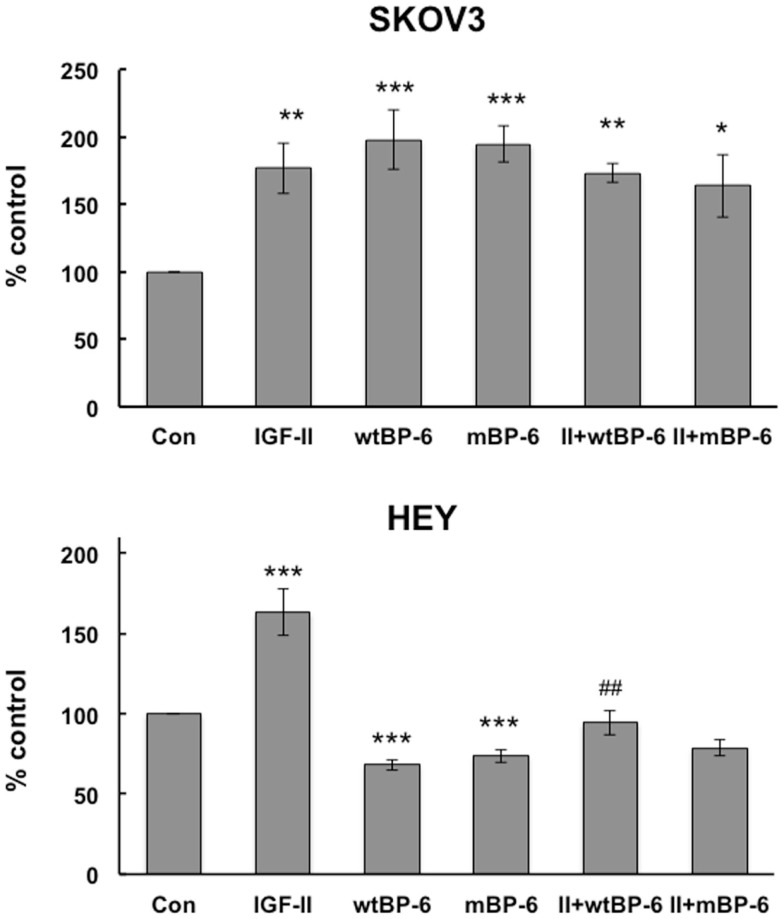
**Effects of IGF-II and IGFBP-6 on SKOV3 and HEY cell migration**. Cells were placed in the upper well of a microchemotaxis chamber, and migration across a membrane into the lower chamber containing SFM ± IGF-II (100 ng/ml), wt and/or mIGFBP-6 (1 μg/ml) in the lower wells was measured. Results are shown as a percentage of control (mean ± SEM, *n* = 4–5 independent experiments). *p < 0.05, **p < 0.01, ***p < 0.001 vs. Con; ^##^*p* < 0.01 vs. wtIGFBP-6.

Initially, the effects of IGF-II and wt or mIGFBP-6 on proliferation of ovarian cancer cells were studied. However, none of these proteins had any effect on proliferation (results not shown). Cell migration was then studied using a microchemotaxis chamber. SKOV3 cell migration was increased by IGF-II (177 ± 19% of control, *p* < 0.01), wtIGFBP-6 (198 ± 22% of control, *p* < 0.001), and mIGFBP-6 (195 ± 13% of control, *p* < 0.001) (Figure 1). Coincubation of IGF-II with either wt or mIGFBP-6 had no additional effect on migration of these cells. Collectively, these results suggest that the effect of IGFBP-6 was IGF-independent.

Insulin-like growth factor-II also increased HEY cell migration to 155 ± 13% of control (*p* < 0.001) but wtIGFBP-6 and mIGFBP-6 each decreased migration to 62 ± 5 and 66 ± 3% of control, respectively (*p* < 0.001) (Figure 1). Coincubation of IGF-II reversed the effect of wtIGFBP-6 to 95 ± 7% of control (*p* < 0.01 wtIGFBP-6 vs. wtIGFBP-6 + IGF-II). In contrast, coincubation of IGF-II had no significant effect on the mIGFBP-6-induced decrease in migration. These results suggest that the effect of IGFBP-6 was IGF-dependent to some extent in these cells.

OVCAR-3 cells did not migrate under basal conditions or after incubation with IGF-II or IGFBP-6 (results not shown) and were not studied further.

### IGFBP-6 increases MAP kinase activation in ovarian cancer cells

**Figure 2:**
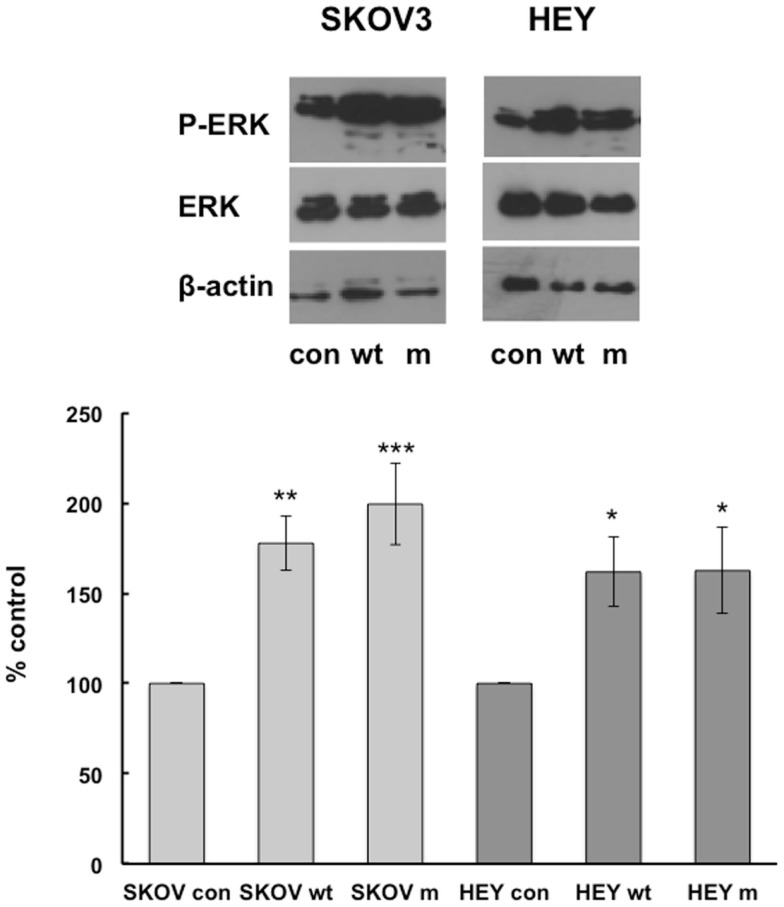
**IGFBP-6 increases phosphorylation of ERK in SKOV3 and HEY cells**. Following serum starvation overnight, SKOV3 and HEY cells were treated without (con) or with wt or mIGFBP-6 (1 μg/ml) for 10 min. Cell lysates were subjected to Western blotting analysis using antibodies against phospho-ERK. Blots were stripped and reprobed with total ERK. β-actin was used as a protein loading control. (Upper): representative blots. (Lower): extent of phosphorylation in response to wtIGFBP-6 and mIGFBP-6 was calculated as the ratio of phospho/total ERK. Results are shown as mean ± SEM of seven to nine independent experiments and expressed as a percentage of control. *p < 0.05, **p < 0.01, ***p < 0.001 vs. Con.

We have previously shown that MAP kinase pathways are involved in IGFBP-6-induced rhabdomyosarcoma cell migration (9, 10), so we compared activation of these pathways in HEY and SKOV3 cells. Wt and mIGFBP-6, respectively, increased ERK phosphorylation in SKOV3 cells to 178 ± 15% (*p* < 0.01) and 199 ± 22% (*p* < 0.001) of control (Figure 2). Similarly, wt and mIGFBP-6, respectively, increased ERK phosphorylation in HEY cells to 162 ± 19% (*p* < 0.05) and 163 ± 24% (*p* < 0.05) of control.

**Figure 3:**
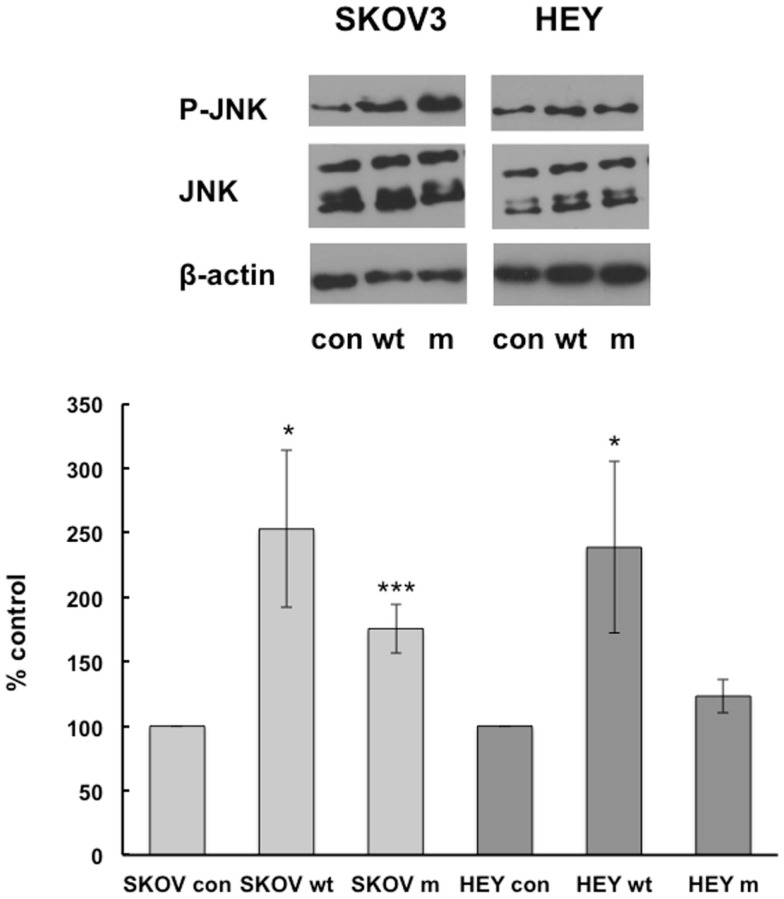
**IGFBP-6 increases phosphorylation of JNK in SKOV3 and HEY cells**. Following serum starvation overnight, SKOV3 and HEY cells were treated without (con) or with wt mIGFBP-6 (1 μg/ml) for 10 min. Cell lysates were subjected to Western blotting analysis using antibodies against phospho-JNK. Blots were stripped and reprobed with total JNK. β-actin was used as a protein loading control. (Upper): representative blots. (Lower): extent of phosphorylation in response to wtIGFBP-6 and mIGFBP-6 was calculated as the ratio of phospho/total ERK. Results are shown as mean ± SEM of five to six independent experiments and expressed as a percentage of control. *p < 0.05, ***p < 0.001 vs. Con.

wtIGFBP-6 also increased JNK phosphorylation in SKOV3 and HEY cells to 253 ± 61% (*p* < 0.05) and 239 ± 66% (*p* < 0.05) of control, respectively (Figure 3). In contrast, mIGFBP-6 significantly increased JNK phosphorylation to 176 ± 19% (*p* < 0.001) of control in SKOV3 cells but not in HEY cells (123 ± 13% of control, NS).

Phosphorylation of p38 MAPK was not detected in either cell line under basal conditions or following incubation with wt or mIGFBP-6 (results not shown).

### Effects of ERK and JNK pathway inhibitors on IGFBP-6-induced migration in ovarian cancer cells

PD98059, an inhibitor of ERK1/2 activation, and SP600125, a JNK inhibitor, were used to study the roles of MAPK pathways in IGFBP-6-induced ovarian cancer cell migration. These inhibitors were shown to, respectively, decrease ERK and JNK phosphorylation in each cell line at the concentration used (30 μM, results not shown).

**Figure 4:**
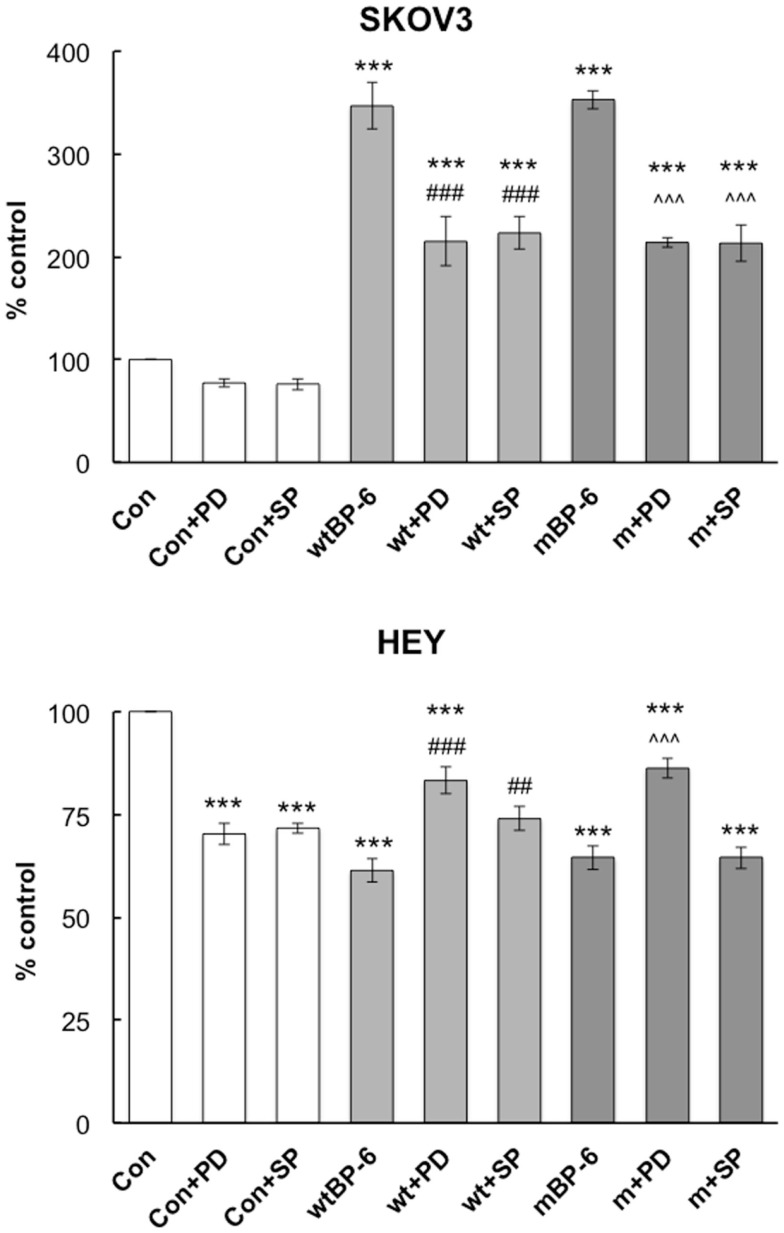
**Effects of ERK and JNK inhibitors on IGFBP-6-induced alterations in SKOV3 and HEY cell migration**. Experiments were performed in serum-free medium in the absence or presence of wt or mIGFBP-6 (1 μg/ml) in the lower wells and inhibitors (PD98059 and SP600125, 30 μM) in both the upper and lower wells of the chemotaxis chamber. Results are shown as a percentage of control (mean ± SEM of three independent experiments). ***p < 0.001 vs. Con; ^##^*p* < 0.01, **^###^***p* < 0.001 vs. wtIGFBP-6; ˆˆˆ*p* < 0.001 vs. mIGFBP-6.

In SKOV3 cells, neither inhibitor had a significant effect on basal migration (Figure 4). PD98059 partially inhibited wtIGFBP-6-induced migration from 347 ± 22 to 215 ± 24% of control (*p* < 0.001). This inhibitor had a similar effect on mIGFBP-6-induced migration (214 ± 5 vs. 353 ± 9% of control, *p* < 0.001). SP600125 also partially abrogated SKOV3 cell migration induced by wtIGFBP-6 (223 ± 16 vs. 347 ± 22% of control, *p* < 0.001) and mIGFBP-6 (203 ± 18 vs. 353 ± 9% of control, *p* < 0.001).

In HEY cells, both inhibitors decreased basal migration by ~30% (*p* < 0.001, Figure 4). Despite this, PD98059 partially restored migration in the presence of wtIGFBP-6 (83 ± 3 vs. 61 ± 3% of control, *p* < 0.001) and mIGFBP-6 (86 ± 2 vs. 65 ± 3% of control, *p* < 0.001). SP600125 also partially restored migration in the presence of wtIGFBP-6 (74 ± 5 vs. 61 ± 3% of control, *p* < 0.01), although the effect was less potent than that of PD98059 (*p* < 0.05). SP600125 had no effect on mIGFBP-6-induced inhibition of HEY cell migration (64 ± 3 vs. 65 ± 3% of control, NS).

Since both inhibitors had partial effects on IGFBP-6-induced changes in migration in each cell line, the effects of a combination of the two inhibitors were studied to determine whether it would completely abolish migration. However, the combination resulted in significant toxicity in both cell lines (results not shown).

## Discussion

The findings of this study showed that IGFBP-6 increased migration of SKOV3 ovarian cancer cells in an IGF-independent manner, which is similar to our previous findings in rhabdomyosarcoma and colon cancer cells (9–11). However, in contrast to these cell lines, IGFBP-6 inhibited migration of HEY ovarian cancer cells. IGF-II reversed the inhibitory effects of wt but not mutant IGFBP-6 in these cells, suggesting that IGFBP-6 inhibited migration by both IGF-dependent and IGF-independent mechanisms. Wt and mutant IGFBP-6 increased ERK and JNK phosphorylation in both cell lines, whereas mutant IGFBP-6 only increased ERK phosphorylation in HEY cells. Inhibitors of ERK and JNK partially inhibited the migratory effects of wt and mIGFBP-6 in SKOV3 cells, whereas the ERK inhibitor partially restored wt and mIGFBP-6-induced inhibition of HEY cell migration. The JNK inhibitor had a lesser effect on the actions of wtIGFBP-6 and no significant effect on the actions of mIGFBP-6 in HEY cells. The latter is consistent with the lack of effect of mIGFBP-6 on JNK phosphorylation in these cells.

We previously showed that IGFBP-6-induced migration of rhabdomyosarcoma cells is dependent on activation of MAP kinase pathways. In RD rhabdomyosarcoma cells, IGFBP-6 transiently increased phosphorylation of p38 MAPK, whereas ERK was constitutively activated and IGFBP-6 had no further effect (9). Specific p38 and ERK inhibitors completely abolished mIGFBP-6-induced RD cell migration as did p38 knockdown. In contrast, the JNK pathway appeared to have no role in IGFBP-6-induced migration of these cells. The pattern of MAPK activation was different in Rh30 rhabdomyosarcoma cells, which derive from a distinct tumor subtype (10). IGFBP-6 increased phosphorylation of ERK and JNK, but had no effect on p38 phosphorylation in these cells. ERK inhibition abolished mIGFBP-6-induced migration as did a p38 inhibitor, despite IGFBP-6 not inducing p38 phosphorylation. A JNK inhibitor also partially inhibited IGFBP-6-induced Rh30 cell migration. These and other findings suggested that crosstalk among MAP kinase pathways played an important role in IGFBP-6-induced cell migration.

MAP kinases are also implicated in ovarian cancer cell migration. MAP4K4 increased SKOV3 cell migration via activation of JNK but not ERK or p38 MAP kinases (23). ERK and JNK were also implicated in mesothelin-stimulated migration of ovarian cancer cells (24), whereas JNK but not ERK or p38 MAP kinase was involved in GnRH-induced ovarian cancer migration (25). Interestingly, the latter study included OVCAR-3 cells, which did not migrate under any conditions in the current study; the reasons for the different responses are unclear but may relate to specific tissue culture conditions. Nevertheless, these findings are consistent with the present study showing that MAP kinase inhibition decreases IGFBP-6-induced migration of SKOV3 cells.

Although MAP kinase activation is associated with increased migration of many cancer cell lines, they may also inhibit this process in some circumstances. For example, ERK activation inhibits neutrophil migration whereas p38 MAP kinase enhances it (26). Similarly, ERK activation mediates CXCL1-induced inhibition of airway smooth muscle cell migration (27). These findings are consistent with those of the present study showing that ERK inhibition partially reversed IGFBP-6-induced inhibition of HEY cell migration.

MAP kinase pathways are not the only signaling pathways involved in cell migration. For example, the PI3 kinase/Akt pathway also mediates migration of some cells (28) including ovarian cancer cells (29). We performed preliminary time-course experiments to determine the effects of wt and mIGFBP-6 on Akt phosphorylation. There was no effect in SKOV cells but there appeared to be an increase in HEY cells (results not shown). However, these results were highly variable and do not explain the inhibitory effect of IGFBP-6 on HEY cell migration. Further, we have previously found that Akt was not involved in IGFBP-6-induced rhabdomyosarcoma cell migration (10).

We showed that IGFBP-6-induced migration of rhabdomyosarcoma cells is dependent on binding to prohibitin-2 (11). Knockdown of prohibitin-2 abrogated IGFBP-6-induced migration of these cells without perturbing IGFBP-6-induced phosphorylation of ERK or JNK, suggesting that its effect was downstream or independent of MAP kinases (11). The ovarian cancer cell lines used in the present study express prohibitin-2 (results not shown), so it would be of interest to determine whether this protein is involved in the migratory responses observed in the present study.

Other IGFBPs have been shown to have context-specific differences in IGF-independent actions. For example, IGFBP-3 inhibited or enhanced EGF-dependent proliferation of breast epithelial cells depending on whether or not fibronectin was used to coat tissue culture plates (30). In contrast with the present study, IGFBP-3 also had differential effects on ERK phosphorylation in these breast cells. A number of other studies have suggested that interactions of IGFBP-3 with other modulators including GRP78 and the sphingolipid rheostat may also play a role in these differential responses (31). Future studies of potential interactions between IGFBP-6 and these modulators would be of interest.

In conclusion, this study has shown differential effects of IGFBP-6 on migration of two ovarian cancer cell lines. IGFBP-6-dependent changes in migration of both cell lines were accompanied by MAP kinase pathway activation, so that this cannot explain the opposite direction of the migratory responses. IGFBP-6 inhibits the actions of IGF-II (7) and angiogenesis by an IGF-independent pathway (8), and both of these may contribute to its anti-tumorigenic effects. However, promotion of migration may be pro-tumorigenic, so further studies to determine the mechanisms underlying the differential effects of IGFBP-6 on migration in these cell lines would help in developing an IGFBP-6-based therapeutic for IGF-II-dependent cancers. These studies could include determining differences in the molecular signatures of these cell lines, which may in turn suggest additional potential therapeutic targets and/or biomarkers of tumor aggressiveness. Metastasis is a complex, multistage process, so studying the effects of IGFBP-6 on other aspects such as cell adhesion would also be of interest. Finally, once the determinants of the promigratory actions of IGFBP-6 are understood, combination therapies including an “optimized” IGFBP-6 molecule and an IGF-I receptor inhibitor may synergistically provide dual blockade of the IGF pathway together with beneficial IGF-independent actions of the binding protein.

## Conflict of Interest Statement

The authors declare that the research was conducted in the absence of any commercial or financial relationships that could be construed as a potential conflict of interest.

## Footnotes

^1^http://imagej.nih.gov/ij
